# Precision and genomic medicine for dilated and hypertrophic cardiomyopathy

**DOI:** 10.3389/fcvm.2023.1137498

**Published:** 2023-03-06

**Authors:** Seitaro Nomura, Minoru Ono

**Affiliations:** ^1^ Department of Cardiovascular Medicine, Graduate School of Medicine, The University of Tokyo, Tokyo, Japan; ^2^ Department of Cardiac Surgery, Graduate School of Medicine, The University of Tokyo, Tokyo, Japan

**Keywords:** dilated cardiomyopathy, hypertrophic cardiomyopathy, genetics, single-cell omics, precision medicine

## Abstract

Cardiomyopathy develops through an interaction of genetic and environmental factors. The clinical manifestations of both dilated cardiomyopathy and hypertrophic cardiomyopathy are diverse, but genetic testing defines the causative genes in about half of cases and can predict clinical prognosis. It has become clear that cardiomyopathy is caused not only by single rare variants but also by combinations of multiple common variants, and genome-wide genetic research is important for accurate disease risk assessment. Single-cell analysis research aimed at understanding the pathophysiology of cardiomyopathy is progressing rapidly, and it is expected that genomic analysis and single-cell molecular profiling will be combined to contribute to more detailed stratification of cardiomyopathy.

## Introduction

Thirty years have passed since it became clear that the cause of cardiomyopathy is genetic mutations, and precision and genomic medicine based on an understanding of the pathogenesis of individual patients. In this paper, we will summarize the current findings in advancing genomic medicine for cardiomyopathy, and discuss future perspectives for the development of optimal treatment methods based on molecular pathology and the establishment of more precise stratification integrated with artificial intelligence.

### Genetic architecture of dilated cardiomyopathy

Dilated cardiomyopathy (DCM), characterized by diffuse contractile dysfunction of the left ventricle and dilatation of the left ventricular lumen in the absence of abnormal stress conditions, such as hypertension, valvular disease, and coronary artery disease, and is a progressive disease with poor prognosis with repeated exacerbations of heart failure symptoms (1). It has been found that genetic factors account for the majority of the background of DCM (2). The causative genes encode proteins that are considered important for maintaining cardiomyocyte function, such as Titin (encoded by the *TTN* gene), Lamin A/C (encoded by the *LMNA* gene), and Desmoplakin (encoded by the *DSP* gene). DCM develops when mutations (mainly rare single-nucleotide mutations) occur in these gene regions and abnormal proteins are produced.

Furthermore, recent studies have shown that truncation mutations in the *TTN* gene, the most common DCM causative mutations, can cause alcoholic cardiomyopathy (3), peripartum cardiomyopathy (4), and chemotherapy-related cardiomyopathy (5), suggesting that a second hit caused by environmental factors in addition to genetic factors induces DCM-like contractile dysfunction. Whole-exome sequencing analysis of 5,942 heart failure patients revealed that truncating mutations in the *TTN* gene were enriched in heart failure patients, and DCM-causing gene mutations were found in 3.4% of all heart failure patients (6), suggesting that the genetic factors of DCM are also important in understanding the pathogenesis of heart failure. Recent genetic studies revealed that truncation mutations of the *DSP* gene and the *TTN* gene contribute to the onset of acute myocarditis (7), revealing that the genetic background of DCM can present such diverse pathologies. Whole-exome sequencing analysis of the UK Biobank data revealed that *TTN* gene mutations are associated not only with DCM, but also with heart failure, atrial fibrillation, supraventricular arrhythmia, ventricular arrhythmia, and valvular disease (8).

DCM has been thought to be caused by rare variants of single causative genes, which are extremely rare in the general population but have a strong impact on the disease onset. However, it has recently become clear that combinations of common variants in multiple SNP regions, which are frequent in the general population but have a weak effect on the disease onset, also cause cardiomyopathy (9, 10). Therefore, efficient investigation of both rare and common variants is important for understanding the genetic factors of DCM. Recent international collaborative studies conducted GWAS related to the structure and function of the right ventricle using the UK Biobank data, and scored the polygenic risk score (the sum of the contributions of SNPs to the disease onset) to find that the score can be used to genetically predict the onset of DCM (11, 12). Furthermore, there is also a report that clonal hematopoiesis of indeterminate potential (CHIP) is associated with the development of heart failure (13), and it is interesting to see how CHIP-related somatic mutations are related to germline mutations involved in the development of DCM. In addition, it has been reported that mutations in the enhancer regions of the *LMNA* and *MYH7* genes are involved in the development of DCM (14), and genome-wide genetic research is important for accurate disease risk assessment.

The clinical manifestations of DCM vary widely in terms of heart failure symptoms, degree of left ventricular systolic dysfunction, age of onset, concomitant arrhythmia, and treatment response. These diverse pathologies may be explained by differences in causative gene mutations, and studies are underway to clarify the relationship between genetic factors and clinical phenotypes (genotype-phenotype association). A genome cohort study revealed that DCM cases with pathogenic or likely pathogenic mutations in known causative genes show a poorer clinical prognosis compared to cases without these mutations (15).

Integrative analysis of genotypes and clinical features of 120 DCM patients revealed that *TTN* truncation mutations, which account for 12%–25% of DCM, were a favorable prognosis group with reverse left ventricular remodeling (restoration of systolic function) in response to medical therapy, whereas *LMNA* gene mutations, which account for 4%–10% of DCM, were a poor prognosis group without reverse remodeling (16). Furthermore, it was also revealed that patients with *LMNA* mutations were predisposed to develop fatal ventricular arrhythmias as well as severe heart failure (17). Given the extremely high penetrance of *LMNA* mutations (17), mutation detection of this gene is important in the diagnosis of cardiomyopathy. A multicenter genome cohort trial revealed that predictors of life-threatening ventricular arrhythmia in patients with *LMNA* mutations were male sex, nonmissense *LMNA* mutation, first degree and higher atrioventricular block, nonsustained ventricular tachycardia, and left ventricular ejection fraction, which facilitates the choice of candidates for implantable cardioverter defibrillators (18). Furthermore, a recent study reported that not only *LMNA* gene mutations, but also *DSP*, *PKP2*, and *FLNC* gene mutations in DCM cases are likely to cause sudden death and ventricular arrhythmia (19), supporting the usefulness of genetic testing in the clinical testing. Integrated analysis of gene mutations and detailed clinical features revealed that independent predictors of pathogenic mutation-positive were family history of DCM, low electrocardiogram voltage in peripheral leads, skeletal myopathy, absence of hypertension, and absence of left bundle branch block (20). In the future, further progress will be made in research that comprehensively analyzes genotypes and phenotypes.

### Genetic architecture of hypertrophic cardiomyopathy

Hypertrophic cardiomyopathy (HCM) is a group of disorders characterized by hypertrophy of the left or right ventricular myocardium and left ventricular diastolic dysfunction due to cardiac hypertrophy. Mutations in sarcomere genes are considered to be the main cause of HCM (21), and the pathology of HCM differs greatly depending on the presence or absence of sarcomere gene mutations. Patients with sarcomere gene mutations tend to have juvenile onset, familial onset, and predisposition to cardiovascular events (sudden death, fatal ventricular arrhythmias, and heart transplants due to severe heart failure) (22), demonstrating the clinical importance of detection of HCM mutations. An analysis using the UK Biobank data also showed that the presence of sarcomere gene pathogenic variants increases the risk of cardiovascular death and heart failure (23). Among HCM, 7% to 8% of cases progress to the diastolic phase and cause contractile dysfunction. Thin filament sarcomere gene mutations tend to result in decreased left ventricular contractility, and comorbid atrial fibrillation and multiple sarcomere gene mutations are associated with poor prognosis (24). Integrated analysis of the genome, transcriptome, metabolome, and lipidome of HCM patients revealed decreased mitochondrial gene expression, disruption of mitochondrial structure, activation of oxidative stress, and decreased mitophagy in the HCM heart (25), presumably leading to the development of therapeutic methods targeting these molecular mechanisms.

The availability of genetic testing has enabled genetic screening of cardiomyopathy families to identify asymptomatic family members harboring mutations that can cause cardiomyopathy. Lorenzini et al. conducted a 15-year clinical follow-up of cardiomyopathy mutation-positive family members who did not initially display the HCM phenotype, and found that approximately 50% of these family members developed HCM (26). In particular, male sex and an abnormal ECG were found to be associated with a higher risk of developing HCM (26), and combining these clinical presentations with genetic screening is expected to lead to earlier diagnosis of HCM. Because early-onset HCM cases present with early cardiac hypertrophy, left ventricular outflow tract obstruction, and fatal arrhythmia (27), familial genetic screening for presymptomatic diagnosis of cardiomyopathy is expected to advance in the future.

Genetic analysis also increases the possibility of reaching an accurate diagnosis for diseases showing phenotypes similar to HCM, such as Fabry disease and transthyretin cardiac amyloidosis; therefore, the importance of genetic testing in routine clinical practice of cardiomyopathy is clearly increasing. The clinical guideline regarding the interpretation and actionability of genetic testing in cardiomyopathy has also been published by the European Society of Cardiology (28). Furthermore, from the perspective of preventing sudden cardiac death, it is important to diagnose not only cardiomyopathy but also hereditary arrhythmias and cardiac sarcoidosis (29); therefore, it is important to establish a multifaceted diagnostic method including extensive genetic testing.

### Multiomics dissection of cardiomyopathy

Cardiomyopathy develops through an interaction of genetic and environmental factors. Intracellular phenotypes should appear as endophenotypes at the time of disease onset, and identification of such phenotypes will lead to disease stratification and treatment development (30). Recent advances in single-cell RNA-seq have enabled cellular-level molecular profiling of rare patient specimens. Single-cell RNA-seq of the heart from patients with heart failure revealed that activation of DNA damage response and TGF-*β* signaling and downregulation of genes related to mitochondrial metabolism are hallmarks of cardiomyocytes of patients with DCM (31, 32) and that dopamine receptor D1 (DRD1) positive cardiomyocytes are characteristic for DCM patients with fatal ventricular arrhythmias (33). Wang et al. used single-cell RNA-seq to analyze the heart before and after implantation of left ventricular assist devices (LVADs) in patients with severe heart failure to find that the downregulated expression of mitochondrial metabolism-related genes was restored after LVAD implantation (34). Ko et al. showed that measurement of IGFBP7, secreted from DNA damage-positive cardiomyocytes in DCM patients, can estimate the severity of heart failure (32). Nicin et al. conducted single-cell analysis of the heart of patients with DCM of various ages, and found that the proportion of cardiac fibroblasts in the heart increased with age and that expression of genes involved in collagen fibers and proteoglycan modifications was increased with age (35). Verdonschot et al. classified 795 patients with dilated cardiomyopathy into four groups based on clinical information, and performed gene expression analysis of cardiac biopsy specimens of patients belonging to each group (36). The expression of NF-*κ*B signaling- and TNF signaling-related genes was higher in group 2, which is prone to autoimmune disease, compared to group 1, which showed mild cardiac dysfunction. Group 3, which was characterized by concomitant arrhythmia (atrial fibrillation and non-sustained ventricular tachycardia), showed high expression of intercellular adhesion-related genes, and group 4, which showed severe cardiac dysfunction, showed high expression of DNA replication-related genes.

Several recent papers have analyzed a number of cardiomyopathy cases by single-cell RNA-seq and correlated them with their clinical characteristics. Koenig et al. performed single-nucleus RNA-seq using nuclei isolated from the heart of patients with dilated cardiomyopathy, and found the increased population of monocyte-derived inflammatory cells and gene expression patterns characteristic for DCM in fibroblasts, endothelial cells and pericytes (37). Chaffin et al. also performed single-nucleus RNA-seq of the heart of patients with DCM and HCM, found common fibroblast activation pathways in both DCM and HCM, and examined the function of the genes involved in the identified pathways by CRISPR-knockout screen (38). Reichart et al. further performed single-nucleus RNA-seq on the heart of patients with DCM and found that the proportion of cardiomyocytes decreased with DCM, while endothelial and immune cell populations increased (39). Although fibroblasts did not increase in population in DCM, they strongly expressed extracellular matrix-related genes and promoted fibrosis. Endothelin signaling was activated in *LMNA* mutant cardiomyopathy, whereas IL6 signaling was activated in *TTN* mutant cardiomyopathy. Ameen et al. analyzed the human fetal heart by single-cell ATAC-seq (genome-wide analysis of open chromatin regions) to profile cell-type-specific epigenomic changes during development (40). Through integrated analysis with information on genomic mutations that occur in patients with congenital heart disease, they demonstrated that *de novo* mutations contribute to disease development by altering transcription factor binding and downstream gene expression (40).

Spatial omics analysis, which analyzes molecular profiles while preserving the spatial characteristics of cells, has advanced our understanding of the pathogenesis of heart disease. Hill et al. performed integrated multi-omics analysis of the heart from patients with congenital heart disease (CHD), DCM, and HCM, and revealed CHD-specific cell states in cardiomyocytes, which showed characteristics of insulin resistance and increased expression of genes associated with FOXO signaling (41). They also conducted imaging mass cytometry to uncover a spatially resolved perivascular microenvironment consistent with an immunodeficient state in CHD and suggest deficient monocytic immunity in CHD, in agreement with the predilection in CHD to infection and cancer (41). Yamada et al. and Calcagno et al. performed spatio-temporal transcriptomic profiling of a mouse myocardial infarction (MI) model, and revealed activation of mechanosensing pathway in cardiomyocytes, characterized by high expression of *Csrp3* and *Flnc*, at the border zone in acute phase after MI (42, 43). Kuppe et al. performed integrative analysis of human cardiac remodeling after MI using single-cell gene expression, chromatin accessibility, and spatial transcriptomic profiling of myocardial tissues from patients with MI and controls (44). They identified a distinct niche of the border zone surrounding the injured myocardium, with a sharp border between injured and uninjured cell types, marked by a gradient of *ANKRD1* and *NPPB* expression. They also uncovered the fibroblast myeloid cellular heterogeneity after MI and identified a distinct cellular dependency between myofibroblasts and activated phagocytic macrophages. In the near future, it is expected that these single-cell molecular profiling techniques will be combined with other omics information to contribute to more detailed stratification of cardiomyopathy.

### Discussion

**Figure 1:**
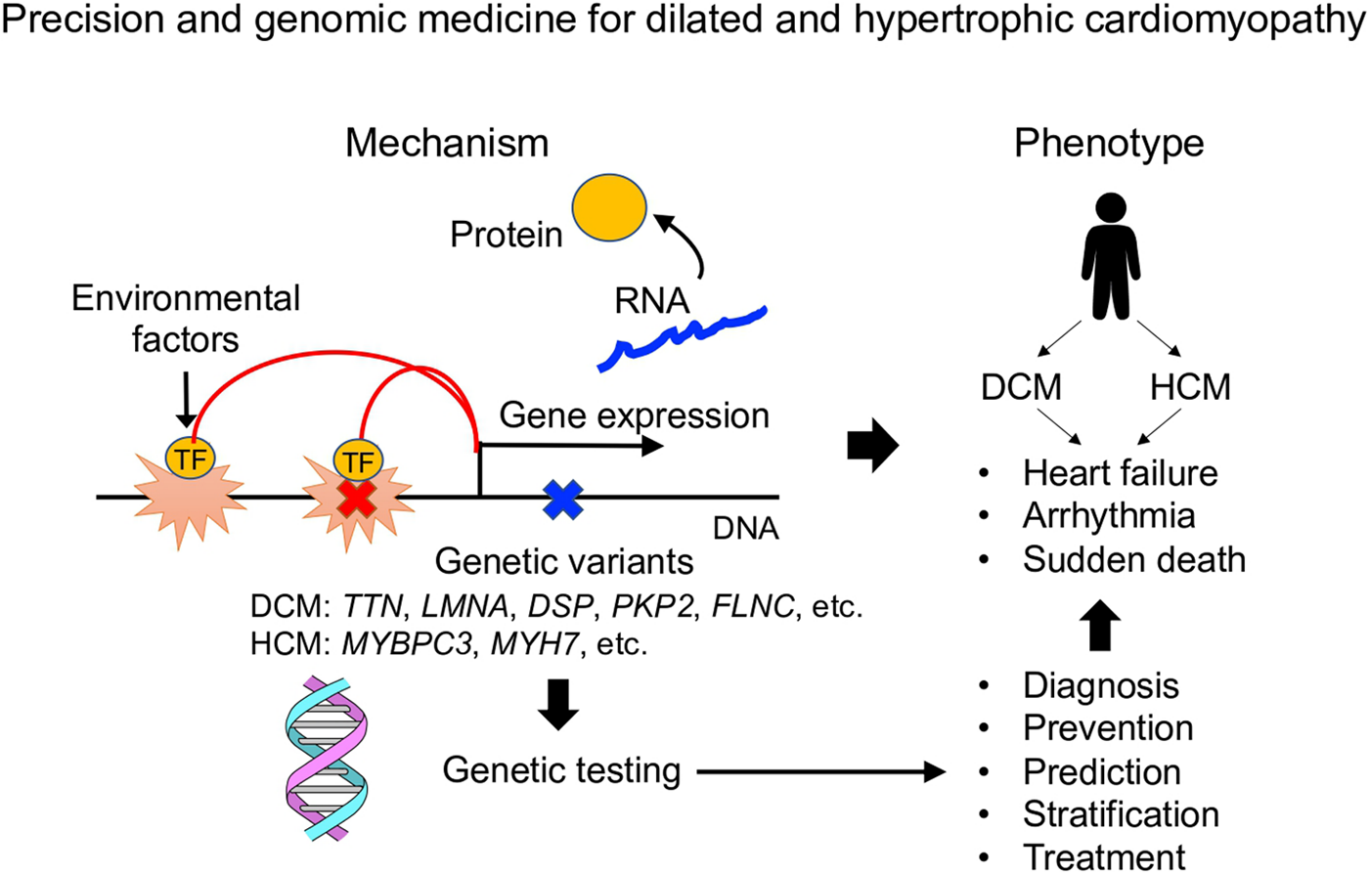
Overview of precision and genomic medicine for dilated and hypertrophic cardiomyopathy.

In this article, we reviewed recent papers that clarified the genetic characteristics of DCM and HCM, and discussed the direction for the development of precision and genomic medicine in cardiomyopathy. Furthermore, we introduced an attempt to understand the molecular pathogenesis of DCM and HCM using single-cell analysis technology, and to promote disease stratification based on the mechanisms. Here, we discuss the challenges in constructing precision medicine based on genetic testing, and the future perspectives of pathophysiological analysis based on the development of single-cell omics analysis technology (Figure 1).

To promote precision medicine based on genetic testing, it is first necessary to generalize the method of mutation analysis and establish a standard genetic testing pipeline. It is also important to develop a method for accurately evaluating the pathogenicity of identified variants. Determining pathogenicity according to the guidelines of American College of Medical Genetics and Genomics (45) is common, but there are also many variants for which pathogenicity cannot be determined at present. It is important to understand the pathogenic significance of these whole-genome variants in detail and improve the accuracy of stratification based on these understandings. In addition, building a reporting system for results including secondary findings and strengthening the system of genetic counseling are also future tasks.

Single-cell omics analysis has made great progress in understanding the pathogenesis of cardiomyopathy, but at this point, the molecular profiling of cell types has only been completed, and it is still far from being connected to medical applications. First, it is important to identify the molecular mechanisms associated with processes that may lead to treatment decision making, and to establish a simple disease stratification method based on these mechanisms. By combining with spatial analysis and comprehensive functional analysis, we need to connect our understanding of RNA expression levels with transcriptional regulation, signaling pathways, cell-cell interactions, and therapeutic interventions. It is also important to promote genome analysis, single cell omics analysis, and medical artificial intelligence in an integrated manner, and to establish a method for integrated analysis of the data obtained by these modalities (46). Furthermore, we must establish effective treatment approaches for each condition stratified by these studies. By moving these things forward, we will be able to advance precision and genomic medicine for cardiomyopathy.

## Author contributions

Paper writing: SN and MO; Supervision: MO. All authors contributed to the article and approved the submitted version.
